# Screening of neuraminidase inhibitory activities of some medicinal plants traditionally used in Lingnan Chinese medicines

**DOI:** 10.1186/s12906-018-2173-1

**Published:** 2018-03-20

**Authors:** Jiawei Liu, Mian Zu, Kaotan Chen, Li Gao, Huan Min, Weiling Zhuo, Weiwen Chen, Ailin Liu

**Affiliations:** 1 Ministry of Education Key Laboratory of Chinese Medicinal Resource from Lingnan, Research Center of Medicinal Plants Resource Science and Engineering, Guangzhou University of Chinese Medicine, 232 Waihuandong Road, Higher Education Mega Center, Guangzhou, 510006 People’s Republic of China; 2 Beijing Key Laboratory of Drug Target Research and Drug Screening, Institute of Material Medica, Chinese Academy Sciences and Peking Union Medical College, 1 Xian Nong Tan Street, Xicheng District, Beijing, 100050 People’s Republic of China

**Keywords:** A (H1N1) influenza virus, Neuraminidase inhibitor, Anti-influenza agents, Medicinal plant, Lingnan Chinese medicines

## Abstract

**Background:**

Neuraminidase (NA) is one of the key surface protein of the influenza virus, and has been established as a primary drug target for anti-influenza therapies. This study aimed to screen bioactive herbal extracts from some medicinal plants traditionally used in Lingnan Chinese Medicines by NA activity high-throughput screening assay.

**Methods:**

One hundred ninety herbal extracts from 95 medicinal plants collected in Guangzhou were screened for their potential inhibitory activities against A (H1N1) influenza neuraminidase, and the most active extracts were further evaluated for their anti-influenza virus activities using virus-induced cytopathic effect (CPE).

**Results:**

Among the tested 190 herbal extracts, 14 extracts inhibited significantly NA activity (IC_50_ < 40 μg/mL), and the extracts **1**–**5**, which were obtained from *Amomurn villosum* Lour, *Melaphis chinensis* (Bell) Baker, *Sanguisorba officinalis* and *Flos Caryophylli*, showed potent inhibitory activity against NA with IC_50_ values ranging from 4.1 to 9.6 μg/mL. Moreover, the most bioactive extracts **1**–**5** were found to protect MDCK cells from A (H1N1) influenza virus infection with very low cytotoxicity to the host cells (EC_50_ values ranged from 1.8 to 14.1 μg/mL, CC_50_ values ranged from 97.0 to 779.2 μg/mL, SI values ranged from 14 to 438). In addition, quantitative RT-PCR analysis showed that the extracts **1**–**5** inhibited viral RNA synthesis in a dose-dependent manner.

**Conclusion:**

We performed in vitro screening of anti-neuraminidase activities of herbal extracts from medicinal plants used in Lingnan Chinese Medicines, and the results indicate that some bioactive extracts are worth further studies to identify the bioactive components responsible for anti-influenza virus activities, to elucidate their modes of action and finally determine their clinical potentials.

## Background

Influenza virus causes an acute contagious respiratory tract infection, which is a major contributor to morbidity and mortality among human population. Historically pandemic flu has caused widespread human deaths, most notably the 1918 “Spanish Flu” (A/H1N1) which killed 25–50 million people worldwide [1]. Novel swine-origin influenza A (H1N1 subtype) virus identified in Mexico in 2009 emerges to spread rapidly worldwide via human-human transmission [2] and led to at least 17,798 deaths in 214 countries. Therefore, pandemic influenza A viruses such as the H1N1subtype becomes a serious global public health problem, which calls for more agents of anti-influenza therapies as possible.

Neuraminidase (NA) is an antigenic glycoprotein on the surface of influenza virus, which takes charge of catalyzing the cleavage of neuraminic acid residues to facilitate the detachment from the host cell surface at the end of the viral replication cycle and suppresses their self-aggregation of the virions [3, 4]. NA plays a critical role for virus replication and spread in infected tissues during infection, and has been well established as a primary drug target for anti-influenza therapies [5, 6]. Some potent NA inhibitors, including oseltamivir, zanamivir, laninamivir and peramivir, have been designed and applied in clinical treatments [7, 8]. Unfortunately, resistance to these NA inhibitors has been extensively reported [9–11]. Therefore, there is a continuing need for developing novel NA inhibitors as anti-influenza agents. Medicinal plants may be a probable source for the discovery of natural NA inhibitors and might provide leads to develop the NA inhibitors [12].

In order to search for novel anti-influenza agents from natural resources, a library of 190 extracts of 95 medicinal plants traditionally used in Lingnan Chinese Medicines were screened for in vitro inhibitory activity against A (H1N1) influenza virus neuraminidase using high-throughput assay. The most active five extracts (**1**–**5**) were selected to further study their action upon the replication of influenza viruses using cytopathic effect (CPE) reduction assay and quantitative RT-PCR analysis. The results showed that these herbal extracts significantly inhibited the NA activity and the replication of influenza viruses, and exhibited very low cytotoxicity to the host cells.

## Methods

### Plant materials

Ninety nine medicinal plants traditionally used in Lingnan Chinese Medicines were collected in Guangzhou in 2009. The identity of the plants samples was verified by Dr. Guangtian Peng (Guangzhou University of Chinese Medicine). Voucher specimens of these materials were deposited for references in the Research Center of Medicinal Plants Resource Science and Engineering, Guangzhou University of Chinese Medicine. The samples were stored in the shade at room temperature and pulverized before use.

### Standard extraction preparation

Dried powdered plants (100 g) were extracted with ethyl acetate (EtOAc, 250 mL × 3) and methanol (MeOH, 250 mL × 3) by ultrasound wave at 40 kHz and 400 W at 45 °C for 30 min, the filtrates were evaporated under vacuum at 45 °C to give the EtOAc and MeOH extracts, respectively. A total of 190 herbal extracts were obtained. A stock solution for each extract was prepared by dissolution to dimethyl sulfoxide (DMSO), 50 mg of each extract was suspended in 1 ml of DMSO ensuing stock concentration of 50 μg/μL. The solutions were filtered by using 0.22 μm filters, and stored at − 20 °C. The concentration of DMSO in test dilutions was restricted to no more than 0.5% (*v*/v) to minimize potential effects of the solvent on enzyme activity and cell growth.

### Neuraminidase, virus and cells

The human influenza virus strains A/PR/8/34 (H1N1) was kindly provided by China Centers for Disease Control, and was used as the source of NA; Madin-Darby canine kidney (MDCK) and A549 cell lines were obtained from the National Center for Pharmaceutical Screening, Institute of Materia Medica, Chinese Academy of Medical Sciences. Madin-Darby canine kidney (MDCK) cells were grown in Dulbecco’s modified Eagle medium (DMEM) containing 10% fetal bovine serum (FBS) at 37 °C and 5% CO_2_ atmosphere. MDCK cells were used for virus infection, and were washed with PBS buffer before infection. 2′-(4-methylunbelliferyl)-*α-D*-acetyl-neuraminic acid (MUNANA), 2-(N-Morpholino)-ethanesulfonic acid (MES) and 3-[4,5-dimethyl-thiazol-2-yl]-2,5-diphenyl tetrazolium bromide (MTT) were purchased from Sigma. DMEM, FBS, and 0.25% trypsin-EDTA were purchased from Gibco. Ribavirin with purity more than 98%, and zanamivir with purity more than 98% were purchased from Sigma (Lot#020 M4003) and Full Land international trade company in Shanghai of China (Lot#091209-005LY), respectively. They were used as references in NA and CPE inhibition assays.

### In vitro screening of plant extracts for NA activity

Inhibition of influenza virus NA activity was determined by a standard fluorimetric method [13, 14] using4-methylumbelliferyl-α-D-N-acetyl-neuraminate (MUNANA) (Sigma) as substrate, in 96-well microplates. The reaction mixture containing the extracts or compounds, and NA enzyme in MES buffer (32.5 mM) and calcium chloride (4 mM, pH 6.5) was incubated for 60 min. After incubation, the reaction was terminated by adding NaOH (34 mM). Fluorescence intensity (M) was quantified with excitation wavelength at 360 nm and emission wavelength at 450 nm. Percentage inhibition was calculated relative to a blank reaction mixture (solvent control) containing virus NA and solvent (% Inhibition = [1-(M_extract_/M_control_)] × 100). The 50% inhibitory concentration (IC_50_) was defined as the concentration of NA inhibitor necessary to reduce NA activity by 50% relative to a blank reaction mixture. IC_50_ values displayed represent the mean of three individual determinations each performed in triplicate assays. Zanamivir (Sigma) was used as the reference compound.

### Cytotoxicity assay

The cytotoxicity of medicinal plant extracts was determined with the MTT (Sigma) method as described previously [15]. Briefly, different concentrations of the extracts and compounds were added to each well of a 96-well culture plate containing a confluent cell monolayer in triplicate, blank medium was used as the control. After incubation at 37 °C in an atmosphere of 5% CO_2_ for 72 h, 12 μL of MTT solution (5 mg/ml in phosphate buffered saline) was added to each well. The plate was further incubated at 37 °C for 3 h to allow formation of formazan product. After removing the medium, 100 μL of DMSO was added to dissolve the formazan crystals. After 15 min, the contents of the wells were homogenized on a microplate shaker. The optical densities (OD) were then determined by measuring absorbance with a microplate spectrophotometer at a wavelength of 540 nm and a reference wavelength of 620 nm. The median cytotoxic concentration (CC_50_) was calculated as the concentration of the constituent that reduced the viable cells to 50% of the untreated control. The maximal non-cytotoxic concentration (MNCC) was defined as the maximal concentration of the sample that did not exert a cytotoxic effect and resulted in more than 90% viable cells.

### CPE reduction assay

The anti-viral activity of the extracts was measured by a virus-induced cytopathic effect (CPE) reduction assay as described previously [14, 16]. Briefly, 100 μL of virus suspension of 200 tissue culture infective dose (TCID_50_/mL) was added to each well of a 96-well culture plate containing confluent a MDCK cells monolayer. After incubation at 37 °C for 2 h, the virus solution was removed, and 100 μL of serial dilutions of the extracts and ribavirin were added to each well of the 96-well culture plates, using the maximal non-cytotoxic concentration (MNCC) as the highest concentration. The plates were incubated at 37 °C in a humidified 5% CO_2_ atmosphere for 48 h, and then the CPE was assessed. The virus-induced CPE was scored as follows: 0 = no CPE, 1 = 0–25% CPE, 2 = 25%–50% CPE, 3 = 50%–75% CPE, and 4 = 75%–100% CPE. Apart from test group, there were control group (treated with FBS-free medium instead of extracts and virus) and model group (treated with FBS-free medium and virus instead of extracts and virus). The CPE inhibition ratios were calculated using the equation: CPE inhibition % = 100 -[(OD_test_-OD_control_) *100/ (OD_model_- OD_control_)]. The OD_test_, OD_model_, and OD_control_ mean the optical density of test group, model group, and control group, respectively. At least three independent experiments with three parallel experiments were performed to determine the mean and SD value.

### Measurement of viral RNA synthesis by quantitative and reverse transcription PCR (qPCR)

A549 cells were grown in RPMI1640 to about 90% confluence and were infected with influenza virus A/PR/8/34 (H1N1) influenza virus at 100 TCID_50_, followed by administration of test extracts for 5 h. To determine the expression level of hemagglutinin (HA) gene mRNA of influenza virus, cells were harvested and the total RNA was extracted by TRIzol (Invitrogen) according to the manufacture’s instruction. The primer sequences which were designed by Primer-BLAST from NCBI for quantitative real-time PCR of influenza virus were 5’-CCTGCTCGAAGACAGCCACAACG-3′ (sense) and 5’-TTCCCAAGAGCCATCCGGCGA-3′ (antisense). The GAPDH were used as internal control of cellular RNAs, with primer sequence of 5′- TGCTCCGAAGGGTGGCCCTTA-3′(sense) and 5′- TGCGTGTTTCCAGAGCCGTGC-3′(antisense). The total RNA was reverse transcribed into cDNA using the TransScript First-Strand cDNA Synthesis SuperMix (TransGen Biotech, Beijing, China). The cDNA was used as template for real-time PCR conducted by SsoFast EvaGreen PCR 2 × master mix (Bio-Rad) using CFX 96 Realtime PCR system (Bio Rad location) according to the manufacture’s protocol. The data was analyzed using the mode for normalised expression (2^-ΔΔCq^).

### Statistical analysis

Statistical analysis was performed using the Student’s unpaired t-test. The results were presented as mean ± S.D. (*n* = 3). ^*^p < 0.05 and ^**^p < 0.001 indicate a statistically significant difference as compared to the untreated control.

## Results

NA has been validated as one of the most important targets to screen the drugs of anti-influenza virus. We first examined the ability of 190 organic extracts from 95 medicinal plants to inhibit NA activity by in vitro screening assay. Zanamivir was used as a positive control, its IC_50_ value to NA inhibition was 0.05 μg/mL. 14 extracts were found to effectively inhibit the NA activity at the concentration of 40 μg/mL. Among them, 5 extracts exhibited potent inhibition of NA activity, 9 extracts exhibited moderate NA inhibitory activity with IC_50_ values ranged from 4.1 to 37.3 μg/mL. The bioactive extracts and their NA inhibition activity were summarized in Table 1. The highest activity was demonstrated by MeOH extracts of *Melaphis chinensis* (**1**) and *Amomurn villosum* Lour (**2**) with IC_50_ = 4.1 and 4.9 μg/mL, respectively. Significant activity with IC_50_ = 5.0–10 μg/mL was also shown by MeOH extract of *Sanguisorba officinalis* (**3**), EtOAc extract of *Melaphis chinensis* (**4**) and MeOH extract of *Flos Caryophylli* (**5**). While other plant extracts (**6**–**14**) showed a moderate inhibitory activity on NA with the IC_50_ values ranging from 20.3 to 37.3 μg/mL. These results demonstrated that these plant extracts possessed significant inhibitory activities against influenza virus NA and the most active extracts **1**–**5** were then selected to further study their effects on the replication of influenza virus.

**Table 1:** Inhibitory activities of Chinese herbs extract on A(H1N1) influenza virus neuraminidase

No.	Positive control and Botanical name	Botanical part	Extract	Inhibition (%)^a^	IC_50_^b^	Voucher No.
–	Zanamivir	–	–	99.8	0.05	–
1	*Melaphis chinensis* (Bell)Baker	cecidium	MeOH	103.6	4.1	MCB091101
2	*Amomurn villosum* Lour.	fruit	MeOH	92.2	4.9	CG20080829
3	*Sanguisorba officinalis L.*	root	MeOH	100.8	5.1	SOL091101
4	*Melaphis chinensis (Bell)Baker*	cecidium	EtOAc	99.3	5.3	MCB091101
5	*Flos Caryophylli*	flowers	MeOH	94.1	9.1	SA091101
6	*Areca catechu Linn*	fruit	MeOH	85.1	19.3	ACL091101
7	*Artemisia capillaries Thunb*	whole plant	MeOH	91.3	19.4	ACT091101
8	*Terminalia chebula Retz*	fruit	EtOAc	78.4	20.3	TCR091101
9	*Duchesnea indica (Andr.) Focke*	whole plant	EtOAc	69.1	23.3	DIF091101
10	*Terminalia chebula Retz.*	fruit	MeOH	68	24.3	TCR091101
10	*Murraya exotica L.*	stem and leaves	MeOH	65.7	28.9	MEL091101
11	*Geranium carolinianum L.*	whole plant	MeOH	64.8	28.9	GCL091101
12	*Polygonum cuspidatum*	rhizome	EtOAc	63.9	29.8	PC091101
13	*Saposhnikovia divaricata (Turez.) Schischk.*	root	EtOAc	53.1	37.3	SDS091101
14	*Callicarpa formosana Rolfe*	fruit	MeOH	47.9	NT^d^	CFR091103
15	*Gardenia jasminoides Ellis*	fruit	MeOH	46.6	NT	GJE091101
16	*Duchesnea indica (Andr.) Focke*	whole plant	EtOAc	46.1	NT	DIF091101
17	*Rosa laevigata Michx.*	stem and leaves	EtOAc	45.8	NT	RLM091103
18	*Euphorbia humifusa Willd. ex Schlecht.*	whole plant	MeOH	43.9	NT	EHW091101
19	*Litchi chinensis Sonn.*	seed	EtOAc	43.9	NT	LCS091101
20	*Punica granatum L.*	fruit peel	MeOH	43.4	NT	PGL091101
21	*Scutellaria baicalensis Georgi*	root	EtOAc	41.3	NT	SBG091101
22	*Amomum villosum Lour.*	fruit	EtOAc	40.5	NT	CG20080829
23	*Geranium carolinianum L.*	whole plant	EtOAc	40.1	NT	GCL091101
24	*Isatis indigotica Fort*	stem and leaves	EtOAc	40.1	NT	IIF091103
25	*Onosma gmelinii Ledeb*	root	EtOAc	40	NT	OGL091101
26	*Houttuynia cordata Thunb*	whole plant	EtOAc	38.5	NT	HCT091101
27	*Altingia chinensis (Champ.) Oliver ex Hance*	stem and leaves	EtOAc	37.3	NT	ACO091103
28	*Pogostemon cablin (Blanco) Bent.*	whole plant	EtOAc	36.7	NT	PCB091101
29	*Polygonum cuspidatum*	rhizome	MeOH	36.1	NT	PC091101
30	*Punica granatum L.*	fruit peel	EtOAc	35.5	NT	PGL091101
31	*Rosa laevigata Michx.*	stem and leaves	MeOH	34.4	NT	RLM091103
32	*Dianella ensifolia (Linn.) Redouté*	fruit	EtOAc	31.5	NT	DER091103
33	*Elsholtzia ciliata (Thunb.) Hyland.*	whole plant	MeOH	31.3	NT	ECH091101
34	*Atractylodes Lancea (Thunb) DC.*	root	EtOAc	30.4	NT	ALD091101
35	*Cynanchum otophyllum Schneid.*	root	EtOAc	29.3	NT	COS091101
36	*Homalocladium platycladum (F. Muell.) Bailey*	whole plant	MeOH	29.1	NT	HPB091101
37	*Cinnamomum cassia Presl*	branch	MeOH	28.9	NT	CCP091101
38	*Elsholtzia ciliata (Thunb.) Hyland.*	whole plant	EtOAc	28.1	NT	ECP091101
39	*Sarcandra glabra (Thunb.) Nakai*	stem and leaves	EtOAc	26.8	NT	SGN091103
40	*Altingia chinensis (Champ.) Oliver ex Hance*	stem and leaves	MeOH	25.8	NT	ACO091103
41	*Litchi chinensis Sonn.*	seed	MeOH	25.5	NT	LCS091101
42	*Phellodendron chinense Schneid*	bark	EtOAC	25.4	NT	PCS091101
43	*Euphorbia humifusa Willd. ex Schlecht.*	whole plant	EtOAc	23.6	NT	EHW091101
44	*Glycyrrhiza uralensis Fisch.*	rhizome	EtOAc	23.1	NT	GUF091101
45	*Woodwardia japonica (L. f.) Sm.*	rhizome	MeOH	23	NT	WJS091101
46	*Ardisia japonica (Thunb) Blume*	whole plant	MeOH	22.7	NT	AJB091101
47	*Cinnamomum cassia Presl*	branch	EtOAc	22.7	NT	CCP091101
48	*Equisetum hyemale L.*	whole plant	EtOAc	22.1	NT	EHL091101
49	*Fraxinus rhynchophylla Hance*	bark	EtOAc	22.1	NT	FRH091101
50	*Ardisia japonica (Thunb.) Blume*	whole plant	EtOAc	21.7	NT	AJB091101
51	*Andrographis paniculata (Burm. f.) Nees*	whole plant	EtOAc	20.8	NT	APN091101
52	*Punica granatum Linn.*	stem	EtOAc	20.2	NT	AGL091103
53	*Syzygium aromaticum*	flowers	EtOAc	19.5	NT	SA091101
54	*Artemisia capillaris Thunb.*	whole plant	EtOAc	19.2	NT	ACT091101
55	*Nepeta cataria L.*	whole plant	MeOH	18.9	NT	NCL091101
56	*Lonicera japonica Thunb.*	flowers	MeOH	18	NT	AJT091101
57	*Woodwardia japonica (L. f.) Sm.*	rhizome	EtOAc	17.9	NT	WJS091101
58	*Nepeta cataria L.*	whole plant	EtOAc	17.4	NT	NCL091101
59	*Dendranthema indicum (L.) Des Moul.*	flowers	EtOAc	16.5	NT	DID091101
60	*Senecio scandens Buch. -Ham. ex D. Don*	whole plant	MeOH	16.3	NT	SSB091101
61	*Onosma gmelinii Ledeb*	root	MeOH	15.9	NT	OGL091101
62	*Evodia rutaecarpa (Juss.) Benth.*	fruit	MeOH	15.5	NT	ERB091101
63	*Ligusticum chuanxiong Hort.*	root	MeOH	15.5	NT	LCH091101
64	*Atractylodes Lancea (Thunb.) DC.*	root	MeOH	15.2	NT	ALD091101
65	*Punica granatum L.*	leaves	MeOH	15	NT	PGL091101
66	*Artemisia indices Willd.*	leaves	MeOH	14.8	NT	AIW091101
67	*Serissa japonica (Thunb.) Thunb.*	stem and leaves	EtOAc	14.8	NT	SJT091101
68	*Prunella vulgaris L.*	whole plant	MeOH	14.1	NT	PVL091101
69	*Dicliptera chinensis (L.) Juss.*	whole plant	MeOH	14	NT	DCJ091101
70	*Glycyrrhiza uralensis Fisch.*	rhizome	MeOH	13.7	NT	GUF091101
71	*Platycladus orientalis (L.) Franco*	leaves	EtOAc	13.4	NT	POF091101
72	*Angelica dahurica (Fisch. ex Hoffm.) Benth.*	root	MeOH	13.3	NT	ADB091101
73	*Sarcandra glabra (Thunb.) Nakai*	stem and leaves	MeOH	13.3	NT	SGN091101
74	*Cynanchum otophyllum Schneid.*	root	MeOH	13	NT	COS091101
75	*Clerodendrum fortunatum Linn.*	stem and leaves	EtOAc	12.5	NT	CFL091101
76	*Scutellaria baicalensis Georgi*	root	MeOH	12.2	NT	SBG091101
77	*Sophora flavescens Alt.*	root	MeOH	11.6	NT	SFA091101
78	*Paris verticillata M.Bieb.*	rhizome	EtOAc	11.4	NT	PVM091101
79	*Semiaquilegia adoxoides (DC.) Makino*	whole plant	EtOAc	11.4	NT	SAM091101
80	*Magnolia liliflora Desr.*	flowers	EtOAc	11.3	NT	MLD091101
81	*Albizia julibrissin Durazz.*	flowers	MeOH	NA^c^	NT	AJD091101
82	*Albizia julibrissin Durazz.*	flowers	EtOAc	NA	NT	AJD091101
83	*Andrographis paniculata (Burm. f.) Nees*	whole plant	MeOH	NA	NT	APN091101
84	*Angelica dahurica (Fisch. ex Hoffm.) Benth.*	root	EtOAc	NA	NT	ADB091101
85	*Arctium lappa L.*	seed	MeOH	NA	NT	ALL091101
86	*Arctium lappa L.*	seed	EtOAc	NA	NT	ALL091101
87	*Areca catechu Linn*	fruit	EtOAc	NA	NT	ACL091101
88	*Artemisia argyi Levl. et Van.*	leaves	MeOH	NA	NT	AAL091101
89	*Artemisia argyi Levl. et Van.*	leaves	EtOAc	NA	NT	AAL091101
90	*Artemisia carvifolia Buch. -Ham. ex Roxb.*	whole plant	EtOAc	NA	NT	ACB091101
91	*Artemisia carvifolia Buch. -Ham. ex Roxb.*	whole plant	MeOH	NA	NT	ACB091101
92	*Artemisia indices Willd.*	leaves	EtOAc	NA	NT	AIW091103
93	*Bidens pilosa Linn.*	whole plant	EtOAc	NA	NT	BPL091103
94	*Bidens pilosa Linn.*	whole plant	MeOH	NA	NT	BPL091103
95	*Bupleurum tenue Buch-Ham. ex D. Don*	root	EtOAc	NA	NT	BTB091101
96	*Bupleurum tenue Buch-Ham. ex D. Don*	root	MeOH	NA	NT	BTB091101
97	*Callicarpa formosana Rolfe*	fruit	EtOAc	NA	NT	CFR091103
98	*Clerodendrum fortunatum Linn.*	stem and leaves	MeOH	NA	NT	CFL091103
99	*Clinopodium megalanthum*	seed	EtOAc	NA	NT	CMC091101
100	*Clinopodium megalanthum*	seed	MeOH	NA	NT	CMC091101
101	*Crataegus pinnatifida Bge.*	fruit	MeOH	NA	NT	CPB091101
102	*Crataegus pinnatifida Bge.*	fruit	EtOAc	NA	NT	CPB091101
103	*Dendranthema indicum (L.) Des Moul.*	flowers	MeOH	NA	NT	DID091101
104	*Dendranthema morifolium (Ramat.) Tzvel.*	flowers	EtOAc	NA	NT	DMT091101
105	*Dendranthema morifolium (Ramat.) Tzvel.*	flowers	MeOH	NA	NT	DMT091101
106	*Dianella ensifolia (Linn.) Redouté*	fruit	MeOH	NA	NT	DER091103
107	*Dicliptera chinensis (L.) Juss.*	whole plant	EtOAc	NA	NT	DCJ091103
108	*Duchesnea indica (Andr.) Focke*	whole plant	MeOH	NA	NT	DIF091103
109	*Epaltes australis Less.*	whole plant	EtOAc	NA	NT	EAL091101
110	*Epaltes australis Less.*	whole plant	MeOH	NA	NT	EAL091101
111	*Equisetum hyemale L.*	whole plant	MeOH	NA	NT	EHL091101
112	*Euchresta japonica Hook. f. ex Regel*	root	EtOAc	NA	NT	EJH091101
113	*Euchresta japonica Hook. f. ex Regel*	root	MeOH	NA	NT	EJH091101
114	*Eupatorium catarium Veldkamp*	whole plant	MeOH	NA	NT	ECV091103
115	*Eupatorium catarium Veldkamp*	whole plant	EtOAc	NA	NT	ECV091103
116	*Eupatorium fortunei Turcz.*	whole plant	EtOAc	NA	NT	EFT091101
117	*Eupatorium fortunei Turcz.*	whole plant	MeOH	NA	NT	EFT091101
118	*Eupolyphaga seu Steleophaga*	insect	EtOAc	NA	NT	ESS091101
119	*Eupolyphaga seu Steleophaga*	insect	MeOH	NA	NT	ESS091101
120	*Evodia rutaecarpa (Juss.) Benth.*	fruit	EtOAc	NA	NT	ERB091101
121	*Ficus hirta Vahl*	leaves	MeOH	NA	NT	FHV091101
122	*Ficus hirta Vahl*	leaves	EtOAc	NA	NT	FHV091101
123	*Forsythia suspensa (Thunb.) Vahl*	fruit	MeOH	NA	NT	FSV091101
124	*Forsythia suspensa (Thunb.) Vahl*	fruit	EtOAc	NA	NT	FSV091101
125	*Fraxinus rhynchophylla Hance*	bark	MeOH	NA	NT	FRH091101
126	*Gardenia jasminoides Ellis*	fruit	EtOAc	NA	NT	GJE091101
127	*Homalocladium platycladum (F. Muell.) Bailey*	whole plant	EtOAc	NA	NT	HPB091103
128	*Homalomena occulta (Lour.) Schot*	rhizome	MeOH	NA	NT	HOS091101
129	*Homalomena occulta (Lour.) Schot*	rhizome	EtOAc	NA	NT	HOS091101
130	*Houttuynia cordata Thunb*	whole plant	MeOH	NA	NT	HCT091101
131	*Ilex cornuta Lindl*	stem	MeOH	NA	NT	ICL091103
132	*Ilex cornuta Lindl*	stem	EtOAc	NA	NT	ICL091103
133	*Inula japonica Thunb.*	flowers	MeOH	NA	NT	IJT091101
134	*Inula japonica Thunb.*	flowers	EtOAc	NA	NT	IJT091101
135	*Isatis indigotica Fort*	stem and leaves	MeOH	NA	NT	IIF091103
136	*Ligusticum chuanxiong Hort.*	root	EtOAc	NA	NT	LCH091101
137	*Lobelia chinensis Lour.*	whole plant	MeOH	NA	NT	LCH091101
138	*Lobelia chinensis Lour.*	whole plant	EtOAc	NA	NT	LCL091101
139	*Lonicera confusa (Sweet) DC.*	stem and leaves	MeOH	NA	NT	LCD091103
140	*Lonicera confusa (Sweet) DC.*	stem and leaves	EtOAc	NA	NT	LCD091103
141	*Lonicera japonica Thunb.*	flowers	EtOAc	NA	NT	LJT091101
142	*Lonicera japonica Thunb.*	stem and branch	MeOH	NA	NT	LJT091101
143	*Lonicera japonica Thunb.*	stem and branch	EtOAc	NA	NT	LJT091101
144	*Lycium chinense Mill.*	root bark	MeOH	NA	NT	LCM091101
145	*Lycium chinense Mill.*	Root bark	EtOAc	NA	NT	LCM091101
146	*Magnolia liliflora Desr.*	flowers	MeOH	NA	NT	MLD091101
147	*Melia azedarach L.*	bark	EtOAc	NA	NT	MAL091103
148	*Melia azedarach L.*	bark	MeOH	NA	NT	MAL091103
149	*Murraya exotica L.*	stem and leaves	EtOAc	NA	NT	MEL091103
150	*Mussaenda pubescens Ait. f.*	stem and leaves	EtOAc	NA	NT	MPA091103
151	*Mussaenda pubescens Ait. f.*	stem and leaves	MeOH	NA	NT	MPA091103
152	*Paris verticillata M.Bieb.*	rhizome	MeOH	NA	NT	PVM091101
153	*Perilla frutescens (L.) Britt.*	flowers	EtOAc	NA	NT	PFB091103
154	*Perilla frutescens (L.) Britt.*	flowers	MeOH	NA	NT	PFB091103
155	*Peucedanum praeruptorum Dunn*	root	EtOAc	NA	NT	PPD091101
156	*Peucedanum praeruptorum Dunn*	root	MeOH	NA	NT	PPD091101
157	*Phellodendron chinense Schneid*	bark	MeOH	NA	NT	PCS091101
158	*Phytolacca acinosa Roxb.*	root	EtOAc	NA	NT	PAR091101
159	*Phytolacca acinosa Roxb.*	root	MeOH	NA	NT	PAR091101
160	*Pinellia ternata (Thunb.) Breit.*	stem	MeOH	NA	NT	PTB091101
161	*Pinellia ternata (Thunb.) Breit.*	stem	EtOAc	NA	NT	PTB091101
162	*Platycladus orientalis (L.) Franco*	leaves	MeOH	NA	NT	POF091101
163	*Pogostemon cablin (Blanco) Bent.*	whole plant	MeOH	NA	NT	PCB091101
164	*Prunella vulgaris L.*	whole plant	EtOAc	NA	NT	PVL091101
165	*Punica granatum L.*	leaves	EtOAc	NA	NT	PGL091103
166	*Punica granatum Linn.*	stem	MeOH	NA	NT	PGL091103
167	*Sanguisorba officinalis L.*	root	EtOAc	NA	NT	SOL091101
168	*Saposhnikovia divaricata (Trucz.) Schischk.*	root	MeOH	NA	NT	SDS091101
169	*Scaphium wallichii Shott & Endl.*	seed	MeOH	NA	NT	SWS091101
170	*Scaphium wallichii Shott & Endl.*	seed	EtOAc	NA	NT	SWS091101
171	*Semiaquilegia adoxoides (DC.) Makino*	whole plant	MeOH	NA	NT	SAM091101
172	*Senecio scandens Buch-Ham. ex D. Don*	whole plant	EtOAc	NA	NT	SSB091101
173	*Serissa japonica (Thunb.) Thunb.*	stem and leaves	MeOH	NA	NT	SJT091103
174	*Sophora flavescens Alt.*	root	EtOAc	NA	NT	SFA091101
175	*Stemona japonica (Bl.) Miq.*	root	MeOH	NA	NT	SJM091101
176	*Stemona japonica (Bl.) Miq.*	root	EtOAc	NA	NT	SJM091101
177	*Strobilanthes cusia (Ness) W. Ktze.*	stem and leaves	MeOH	NA	NT	SCW091101
178	*Strobilanthes cusia (Ness) W. Ktze.*	stem and leaves	EtOAc	NA	NT	SCW091101
179	*Thlaspi arvense L.*	whole plant	MeOH	NA	NT	TAL091103
180	*Thlaspi arvense L.*	whole plant	EtOAc	NA	NT	TAL091103
181	*Turczaninovia fastigiata (Fisch.) DC.*	flowers	MeOH	NA	NT	TFD091101
182	*Turczaninovia fastigiata (Fisch.) DC.*	flowers	EtOAc	NA	NT	TFD091101
183	*Vitex trifolia L.*	stem and leaves	EtOAc	NA	NT	VTL091103
184	*Vitex trifolia L.*	stem and leaves	MeOH	NA	NT	VTL091103
185	*Wikstroemia indica (Linn.) C. A. Mey.*	whole plant	MeOH	NA	NT	WIC091103
186	*Wikstroemia indica (Linn.) C. A. Mey.*	whole plant	EtOAc	NA	NT	WIC091103
187	*Xanthium sibiricum Patrin ex Widder*	fruit	EtOAc	NA	NT	XSP091103
188	*Xanthium sibiricum Patrin ex Widder*	fruit	MeOH	NA	NT	XSP091103
189	*Zanthoxylum nitidum (Roxb.) DC.*	root	MeOH	NA	NT	ZND091101
190	*Zanthoxylum nitidum (Roxb.) DC.*	root	EtOAc	NA	NT	ZND091101

^a^Percentage inhibition was calculated relative to a blank group containing virus NA but no inhibitors, final concentration at 40 μg/mL; ^b^IC_50_ values represent the concentration that caused 50% NA enzyme activity loss, the average of at least three independent assays, IC_50_ values are in μg/mL. ^c^: not active; ^d^: not test

To validate whether these extracts **1**–**5** that exhibited NA inhibitory activity could protect host cells from influenza virus A (H1N1) infections, the CPE reduction assay was carried out in MDCK cells. The human influenza virus A/PR/8/34 (H1N1) strain was used to infect MDCK cells. Cells were incubated in the presence or absence of the extracts **1–5**, after 48 h of incubation, their CPE reduction activity on virus multiplication was then examined. As shown in Table 2, the extracts **1–5** could protect MDCK cells from the infection of influenza virus A (H1N1), exhibited a drastic reduction of influenza virus-induced CPE. The EC_50_ values of the extracts **1**–**5** ranged from 1.8 to 14.1 μg/mL, similar to the results obtained in NA assays. Among the five extracts, the MeOH extract (**2)** from the fruits of *Amomurn villosum* had excellent CPE activity with very low EC_50_ values of 1.8 μg/mL, this is comparable to that of the positive compound ribavirin (3.2 μg/mL). The viability of MDCK cells incubated in the presence or absence of the extracts was evaluated by MTT assay, the CC_50_ values of the extracts **1**–**5** was found to be from 97.0 to 779.2 μg/mL, suggesting that the extracts protected significantly host cells from influenza virus infection and did not exhibit considerable cytotoxicity against MDCK cells. The maximal non-cytotoxic concentration (MNCC) of the extracts **1–5** were found to be from 30 to 300 μg/mL in MDCK cells. Their therapeutic selective index (SI) in MDCK cells ranged from 14 to 438, and among of them, the SI value of *A. villosum* was highest on basis of its low cytotoxicity and its high CPE effect. These data demonstrated that the extracts **1–5** protected MDCK host cells from viral damage with very low toxicity. Thus, in agreement with that these extracts inhibited NA activities, the extracts **1**–**5** reduced host cell damage caused by the influenza virus A (H1N1) infection.

**Table 2:** Inhibitory activity of Chinese herbs extracts (**1**–**5**) on A(H1N1) influenza virus by CPE assay

Sample No.	EC_50_^a^	CC_50_^b^	MNCC^c^	SI^d^
1	7.7	184.3	30	24
2	1.8	779.2	300	438
3	8.1	478.4	100	59
4	7.2	97.0	30	14
5	14.1	744.3	300	53
Ribavirin	3.2	> 100	—^e^	> 31
Zanamivir	> 90.4	> 1506.0	> 301.2	17

^a^EC_50_: Effective concentration required to protect 50% of cells; ^b^ CC_50_: Median (50%) cytotoxic concentration in MDCK cells; ^c^ MNCC: Maximal non-cytotoxic concentration in MDCK cells, values in μg/mL; ^d^ SI:Selectivity index, CC_50_/EC_50_.^e^: not test

**Fig. 1:**
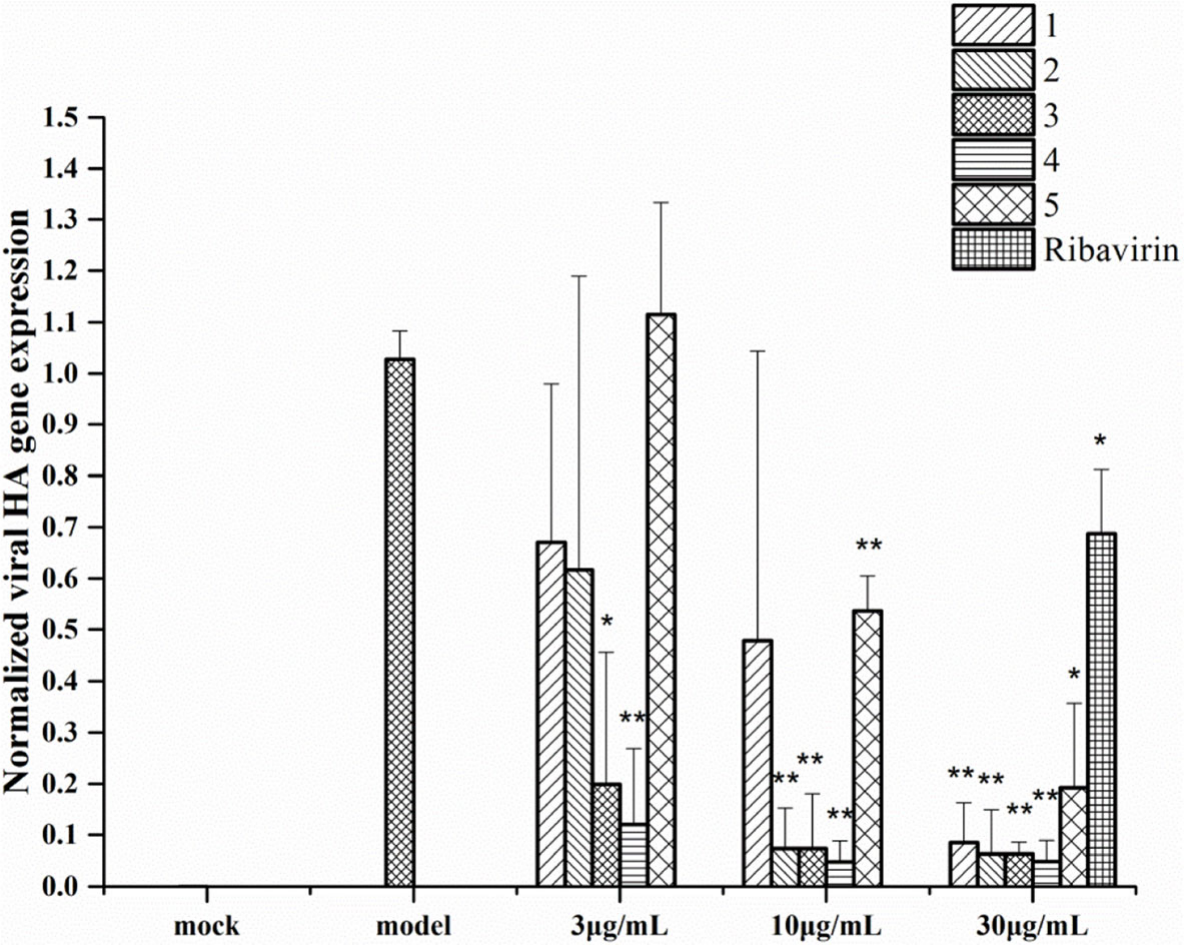
Dose-dependent inhibitory effect of the extracts **1**–**5** on viral RNA synthesis. A549 cells were infected with 100 TCID_50_ influenza H1N1 viruses and treated with different concentrations of the extracts **1**–**5** (3, 10 or 30 μg/mL) and the DMSO (0.03%) for 5 h. The total RNA was extracted and followed by qPCR analysis. To quantify the changes in gene expression, the 2^-ΔΔC(q)^ method was used to calculate relative changes which were normalized to the GAPDH gene and the untreated control (model group, which was set to 1). Value calculated as Mean ± SD of three independent tests, with ^*^ *p* < 0.05 and ^**^p < 0.001, respectively

To further examine whether the protective effect of the extracts**1**–**5** is related with the inhibition of influenza viral replication, total RNA was extracted and subjected to quantitative reverse-transcription PCR in the A/H1N1 virus-infected A549 cells. Our results showed that treatment with the extracts **1**–**5** for 5 h resulted in a substantial reduction in viral RNA expression level in a dose-dependent manner (Fig. 1). All extracts **1**–**5** at the high concentration (30 μg/mL) had significant inhibitory effects on viral RNA expression as compared with untreated control, even more powerful than ribavirin (Fig. 1). The extracts **2**–**5** at medium concentration (10 μg/mL) also demonstrated significant inhibitory effects on viral RNA synthesis. Interestingly, the extracts **3** and **4** at low concentration of 3 μg/mL still significantly inhibited RNA synthesis of influenza viruses. These data indicate that the extracts **1**–**5** could inhibit significantly the replication of influenza viruses in cultures by RT-PCR analysis, which validated their anti-influenza viral activity obtained by CPE reduction assay.

## Discussion

In the course of our screening of NA inhibitors for influenza virus A (H1N1), a total of 190 extracts of 95 medicinal plants traditionally used in Lingnan Chinese Medicines were submitted to in vitro screening for their NA inhibitory activities. Among of them, the organic extracts **1**–**5**, obtained from *Melaphis chinensis*, *Amomurn villosum*, *Sanguisorba officinalis* and *Flos Caryophylli*, were found to significantly inhibit the NA activity (IC_50_ < 10 μg/mL, Table 1) and the replication of influenza virus in a dose-dependent manner (Fig. 1), and exhibited very low cytotoxicity to the host cells with the high selective index (SI) values ranging 14 to 438 (Table 2). Therefore, these Chinese herb extracts might contain bioactive components responsible for anti-influenza virus activity at non-toxic concentration and they could be a promising source of natural NA inhibitors.

It was demonstrated previously that the aqueous extracts of barks, leaves and galls of *Melaphis chinensis* have anti-influenza virus activity and some compounds such as gallotannins isolated from *M. chinensis* are responsible for the anti-influenza virus effect [17]. The presence of such compounds in our EtOAc and MeOH extracts of galls of *M. chinensis* may explain the biological activities seen in our screenings*.*

*Flos Caryophylli* also known as cloves, is considered acrid, warm and aromatic in Traditional Chinese Medicines for the treatment of stomachache, diarrhea and dental pain [18]. It was reported that the hot water extract of *Flos Caryophylli* have been shown to have anti-herpes virus, anti-hepatitis C virus and anti-cytomegalovirus activities in vitro and in vivo, and compounds such as ellagitannin and eugeniin were identified as the bioactive components with anti-virus properties [19]. In the present study, the MeOH extract of *Flos Caryophylli* showed IC_50_ value of 9.1 μg/mL towards NA and EC_50_ value of 14.1 μg/mL against influenza virus. In our latest phytochemical study on the MeOH extract of *Flos Caryophylli* [14], a bioassay-guided isolation led to identification of ten flavonoids, seven tannins and two chromones as NA inhibitors with IC_50_ values ranging from 8.4 to 94.1 μM. These polyphenolic constituents were found to protect MDCK cells from A(H1N1) influenza infections (EC_50_ = 1.5–84.7 μM) with very low cytotoxicity to the host cells (CC_50_ = 374.3–1266.9 μM)), with selective index (SI) ranging from 7 to 297 [14].

The roots of *S. officinalis* (Rosaceae) are well-known Chinese herbs officially listed in the Chinese Pharmacopeia and have been used for the treatment of bleeding, diarrhea and burns. Early chemical studies showed that *S. officinalis* synthesize a variety of secondary metabolites, particularly polyphenols, triterpenoids, saponins and flavonoids with specific biological activities such as anti-asthmatic, anti-bacterial, anti-cancer and anti-inflammation [20–25]. A variety of flavonoids, saponins and polyphenols isolated from medicinal plant have been studied extensively and exhibited anti-influenza activities [12]. The MeOH extract of *S. officinalis* showed strong activities towards NA (IC_50_: 5.1 μg/mL) and against influenza virus (EC_50_: 8.1 μg/mL). The anti-influenza activity may be due to the presence of flavonoids and polyphenols in the MeOH fraction.

The fruits of *A. villosum* (Zingiberaceae) were consumed widely as popular cooking spices in East Asian countries and have been traditionally used as a medicine to treat various digestive disorders [26]. The volatile oils of the fruits of *A. villosum* were shown to be the major components and suggested to be responsible for the different biological activities such as analgesic, anti-oxidation and anti-inflammation [27]. In this study, the MeOH extract of the fruits of *A. villosum*was shown to significantly inhibit NA activities (IC_50_: 4.9 μg/mL) and protect the host cells from CPE damage (EC_50_: 1.8 μg/mL) without cytotoxicity, and its therapeutic selective index (SI) is 439 in MDCK cell culture.

In this study, we limit our study on EtOAc and MeOH extracts of medical plants since bioassay-guided isolation of neuraminidase inhibitors in aqueous extracts remains a challenging task for us. However, this may decrease the risk of false-positive results in the enzyme-based screening caused by some interfering components present within aqueous extracts. Future study will try to improve the screening methods on aqueous extracts that may also contain active components with anti-neuraminidase activity.

## Conclusion

We carried out the in vitro screening of anti-neuraminidase activity of 190 herbal extracts from 95 medicinal plants traditionally used in Lingnan Chinese Medicines. Among the tested extracts, 5 extracts, obtained from *Amomurn villosum*, *Melaphis chinensis*, *Sanguisorba officinalis* and *Flos Caryophylli*, showed potent NA inhibitory activity. Comprehensive literature survey revealed that no study has been reported on the effects of the organic extracts of *A. villosum* and *S. officinalis* on anti-influenza virus activities and small-molecule NA inhibitors from these extracts have not been chemically identified yet. Further studies are underway to isolate bioactive components of these extracts by bioassay-guided fractionation, and to explore their antiviral mechanisms and finally determine their clinical potentials.

## Abbreviations

CPE: Cytopathic effect
HA: Haemagglutinin
HHDP: Hexahydroxydiphenoyl
MDCK: Madin-Darby canine kidney
MNCC: Maximal non-cytotoxic concentration
MTT: 3-[4,5-dimethyl-thiazol-2-yl]-2,5- diphenyl tetrazolium bromide
MUNANA: methylumbelliferyl-α-D-N-acetylneuraminate
NA: Neuraminidase
SI: Selectivity index.
